# Availability and cost of antifungal therapy in Vietnam: A 5-year retrospective study

**DOI:** 10.1093/mmy/myaf028

**Published:** 2025-04-01

**Authors:** Yee Chin Kwang, Ha Thuy Nguyen, Jan-Willem Alffenaar, Justin Beardsley, Vu Quoc Dat

**Affiliations:** Department of Pharmacy, Wagga Wagga Base Hospital, Wagga Wagga, Australia; Hanoi Medical University Hospital, Hanoi Medical University, Hanoi, Vietnam; Department of Infectious Diseases, Hanoi Medical University, Hanoi, Vietnam; School of Pharmacy, University of Sydney, Sydney, Australia; Department of Pharmacy, Westmead Hospital, Sydney, Australia; Sydney Infectious Diseases Institute, University of Sydney, Sydney, Australia; Sydney Infectious Diseases Institute, University of Sydney, Sydney, Australia; Infectious Disease Department, Westmead Hospital, Sydney, Australia; Hanoi Medical University Hospital, Hanoi Medical University, Hanoi, Vietnam; Department of Infectious Diseases, Hanoi Medical University, Hanoi, Vietnam; Sydney Infectious Diseases Institute, University of Sydney, Sydney, Australia

**Keywords:** antifungal, access, availability, cost, vietnam

## Abstract

Access to antifungal agents for the treatment of invasive fungal infections (IFIs) varies significantly between countries. Limited access or high cost may contribute to the burden of IFIs. We aimed to investigate the availability and cost of antifungal treatment for IFIs in Vietnam. Procurement data from 2018 to 2022 was collected from the Drug Administration of Vietnam website. We calculated the cost per defined daily dose (DDD) and identified the manufacturing countries. We explored the pharmacotherapy cost of the four major IFIs if first-line agents were used in accordance with the Vietnam 2021 antifungal prescribing guideline. We also estimated the treatment expenditure in 2020 based on the estimated disease burden previously published and suggested cost-saving measures. At least 57.6 million USD was spent on 15.5 million DDD of antifungals in 5 years. Seven systemic antifungal agents were available in Vietnam. Caspofungin and micafungin were the least used but most expensive, whereas fluconazole and itraconazole were the most consumed but cheapest antifungals. Vietnam manufactured 70% of azole antifungals and relied on imports for the remaining antifungals consumed. We estimated the first-line pharmacological treatment for the estimated cases of four IFIs in 2020 to cost at least 209.1 million USD, which exceeded the actual spend in 2020. We discovered that antifungal agents for IFIs impose a substantial economic burden on Vietnam’s healthcare system. We highlight the need for cost-effectiveness studies of expensive first-line medications. Efforts to mitigate this economic burden should include antifungal stewardship, prevention of IFIs, and sourcing from cost-effective manufacturers.

## Introduction

Invasive fungal infections (IFIs) affect more than 6.55 million people annually and are often associated with high rates of disability and mortality.^1^ Populations at risk include people who are immunocompromised or have underlying comorbidities such as human immunodeficiency virus/acquired immunodeficiency syndrome (HIV/AIDS), diabetes mellitus, critical illness, solid organ transplantation, tuberculosis, or chronic obstructive pulmonary disease.^2^

Ready access to antifungal agents, in terms of both availability and affordability, is key to successful outcomes for these patients. The World Health Organization (WHO) has included amphotericin B, clotrimazole, fluconazole, flucytosine, griseofulvin, itraconazole, nystatin, voriconazole, caspofungin, and micafungin on the essential medicines list (EML).^3^ However, even these life-saving medications are not available in all countries, and high costs can be a further hindrance to access. A study by Kneale et al. in 2016, covering 159 countries with a population greater than 1 million, found a wide variability in the access, availability, and costs of antifungal agents.^4^ For example, amphotericin B was found to be unavailable in 42 of 155 (27.1%) countries, whereas the daily cost of flucytosine varied from $4.60 to $1409 per 5-g oral dose in countries where it was available.^4^ Due to cost, more expensive agents such as echinocandins and voriconazole may not be widely used in resource-limited countries despite being the first-line agents in international consensus guidelines for the treatment of invasive candidiasis and invasive aspergillosis, respectively.^5,6^

Vietnam is a lower-middle-income country with a population of about 100 million.^7^ In 2020, it was estimated as having a high burden of serious fungal infections, with over 2.3 million cases.^7^ Despite this significant burden, fungal diseases receive less attention than bacterial and viral infections, and robust epidemiological data are lacking.^8^

As the cost and availability of antifungal agents and fungal disease prevalence vary among countries, there is a need to investigate the availability and costs of antifungal agents in Vietnam and to estimate their corresponding financial burden in order to inform public health policy and practice tailored to local needs.^4^ The primary aims of this study were to investigate the availability, costs, and sources of antifungal agents in Vietnam by analysing the annual consumption and expenditure over a 5-year period from 2018 to 2022. The secondary aims were to calculate the cost of different treatment regimens for the most common IFIs and to estimate the national medication costs of antifungal therapy of IFIs in Vietnam. The five major fungal species of concern in Vietnam are *Candida, Aspergillus, Cryptococcus, Talaromyces*, and *Pneumocystis* according to the Guideline for the Diagnosis and Treatment of Invasive Fungal Infections published by the Ministry of Health of Vietnam in 2021.^9^*Pneumocystis* was excluded from this study because the infection is treated with trimethoprim-sulfamethoxazole instead of antifungals.

## Methods

### Healthcare system in Vietnam and study approach

Vietnam has 58 provinces and 5 central cities, totalling 63 provincial-level authorities. The public healthcare facilities are divided into four tiers: (i) national or central hospitals, which are under the management of the Ministry of Health; (ii) provincial hospitals, which are governed by the provincial Departments of Health and receive referrals from the provincial population; (iii) district hospitals, which are also managed by the provincial Departments of Health and receive referrals from district health centres and commune health clinics; and (iv) commune health clinics, which focus on primary health services at a commune level.^10^ As of 2020, there were 34 national hospitals, 471 provincial hospitals, 952 district hospitals and health clinics in Vietnam.

In Vietnam, medications are supplied to hospitals by contractors who win tenders through bidding in accordance with government regulations.^11^ Individual hospitals may procure medications independently and directly from the suppliers (decentralised method). Alternatively, medications are procured centrally either by the Ministry of Health at the national level or by the provincial Departments of Health at the provincial level. The procurement needs are estimated based on the consumption in the previous year.^11^ As the procurement regulation requires each health facility to ensure that at least 80% of each medication purchased is consumed, we used the quantity of an antifungal purchased every year as the surrogate indicator of its consumption.^11^

### Data sources and collection

We obtained the annual drug bidding reports submitted by hospitals and provincial Departments of Health to the publicly accessible official website of the Drug Administration of Vietnam, a department within the Ministry of Health that oversees pharmaceuticals in Vietnam.^12^ Data collected included the active ingredient, route of administration, type of antifungal, names of hospitals or Departments of Health, tier of the hospital, year of purchase, quantity, unit price, total cost of purchase, and manufacturing countries.

### Calculation of number of DDD and cost per DDD

We included oral and parenteral antifungal agents used in the treatment of systemic fungal infections as listed in the Anatomical Therapeutic Chemical (ATC) index and excluded those of local or topical fungal infections.^13^

We expressed the consumption of antifungal agents as defined daily doses (DDD), a measurement unit independent of currency, price, and strength.^13^ DDD is the average daily maintenance dose of a drug used for its main indication in an adult weighing 70 kilograms (kg). We used the following DDDs: 35 mg for amphotericin B deoxycholate (J02AA01), 50 mg for caspofungin (J02AX04), 200 mg for fluconazole (J02AC01), 200 mg for itraconazole (J02AC02), 100 mg for micafungin (J02AX05), 300 mg for posaconazole (J02AC04), and 400 mg for voriconazole (J02AC03).

The number of DDD was calculated by multiplying the total doses by the DDD conversion factor.^14^ We calculated the cost per DDD of each antifungal agent by dividing the cost of purchase with the number of DDD. All prices were presented in United States dollars (USD) after conversion from Vietnamese dong (VND) using the average yearly official exchange rate of the World Bank as of the 30th of December 2022 (1 USD = 23 365 VND).

### Estimation of cost of IFI treatment

We estimated the costs of each course of antifungal treatment for the main IFIs caused by *Candida, Aspergillus, Cryptococcus*, and *T. marneffei*.^7^ The Guidelines for Diagnosis and Treatment of Invasive Fungal Infections published by the Vietnam Ministry of Health in 2021 were referred to for recommended treatment duration and choice of antifungal agents.^9^ The costs of treatment were compared between different lines of treatment. We also estimated the cost that would have been invested by Vietnam in each treatment in 2020 based on the estimated fungal disease burden in that year.^7^

### Ethics approval

Ethics approval was not required, as this study only used publicly available data. No individual patient data were used.

## Results

A total of 261 procurement units submitted at least 1 year of data for at least one antifungal agent to the Drug Administration of Vietnam from 2018 to 2022. These procurement units consisted of 32 national hospitals, 59 provincial department of health, 109 provincial hospitals, and 61 district hospitals. Out of these, a mere ≤5 procurement units submitted the data of at least one antifungal agent every year (Supplementary Table 1).

### Availability, costs, and consumption of antifungals in Vietnam

As of 2022, there are seven antifungal agents available for the therapy of systemic IFIs in Vietnam. These antifungal agents can be categorised into three classes, namely polyenes (amphotericin B deoxycholate), azoles (fluconazole, itraconazole, voriconazole, and posaconazole), and echinocandins (caspofungin and micafungin). Micafungin was only introduced to the country in 2021.

Table 1 summarised the expenditure and the number of DDD of each antifungal agent in Vietnam from 2018 to 2022. The detailed breakdown of the consumption and expenditure contributed by centralised bidding as well as national, provincial, and district hospitals is shown in Supplementary Table 2.

**Table 1.** Expenditure (in United States dollar, USD) and number of defined daily dose (DDD) of antifungal agents in Vietnam from 2018 to 2022.*

	2018	2019	2020	2021	2022	
Amphotericin B deoxycholate	Expenditure (%)	2055 400 (14.0)	1439 200 (15.9)	836 900 (11.2)	989 900 (8.9)	1631 800 (10.7)
	Number of DDD (%)	86 000 (1.5)	42 600 (1.6)	36 900 (1.8)	40 300 (1.4)	69 800 (3.6)
Caspofungin	Expenditure (%)	4691 500 (32.0)	3544 500 (39.2)	3672 300 (49.3)	7025 100 (63.1)	9859 200 (64.5)
	Number of DDD (%)	14 800 (0.3)	12 500 (0.5)	13 800 (0.7)	28 400 (1.0)	41 200 (2.1)
Fluconazole	Expenditure (%)	2626 800 (17.9)	1416 500 (15.7)	1461 200 (19.6)	1324 600 (11.9)	793 600 (5.2)
	Number of DDD (%)	3045 600 (51.2)	1355 600 (51.2)	1096 700 (53.3)	1500 100 (51.9)	1084 700 (56.1)
Itraconazole	Expenditure (%)	4415 000 (30.1)	1329 400 (14.7)	891 300 (12.0)	818 400 (7.4)	502 600 (3.3)
	Number of DDD (%)	2773 600 (46.7)	1198 100 (45.3)	891 300 (43.4)	1300 300 (45.0)	699 200 (36.2)
Micafungin	Expenditure (%)	N/A	N/A	N/A	247 100 (2.2)	1137 900 (7.4)
	Number of DDD (%)	N/A	N/A	N/A	1200 (0.04)	5500 (0.3)
Posaconazole	Expenditure (%)	659 600 (4.5)	647 500 (7.2)	484 600 (6.5)	302 300 (2.7)	334 300 (2.2)
	Number of DDD (%)	19 600 (0.3)	20 200 (0.8)	15 700 (0.8)	9900 (0.3)	11 500 (0.6)
Voriconazole	Expenditure (%)	229 900 (1.6)	672 300 (7.4)	99 600 (1.3)	434 500 (3.9)	1038 700 (6.8)
	Number of DDD (%)	5500 (0.1)	18 100 (0.7)	1900 (0.1)	8400 (0.3)	20 200 (1.1)
Total of all antifungals	Expenditure	14 678 200	9049 500	7445 800	11 141 800	15 297 900
	Number of DDD	5945 200	2647 000	2056 300	2888 500	1932 200

DDD, defined daily dose; N/A, not available.

* The calculated expenditures in the table have been rounded to the nearest 100 USD.

**Figure 1.**
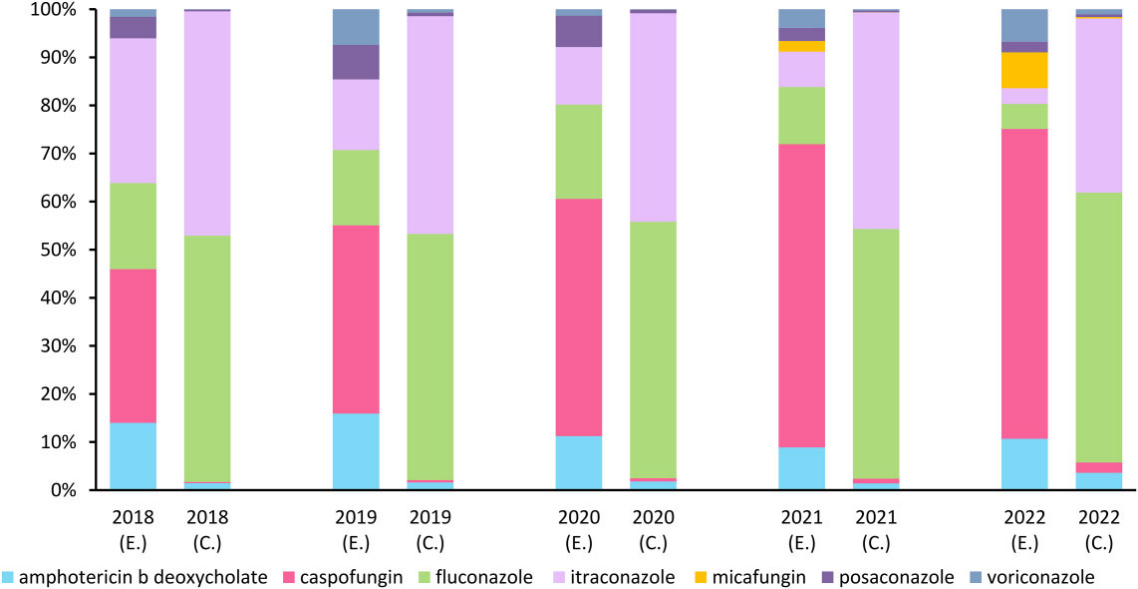
Proportion of the annual expenditure and consumption of individual antifungal agents from 2018 to 2022. E., expenditure; C., consumption (in defined daily dose, DDD).

Over 5 years, Vietnam consumed at least 15.5 million DDD of antifungals, at a cost of at least 57.6 million USD. Figure 1 shows the breakdown of the yearly expenditure and consumption on each antifungal agent from 2018 to 2022 and highlights changing patterns. It is important to note that the trends of expenditure and consumption over time could not be analysed and interpreted from our data because not every procurement unit submitted the data to the Drug Administration of Vietnam every year or for every antifungal agent (Supplementary Tables 3 and 4).

**Figure 2.**
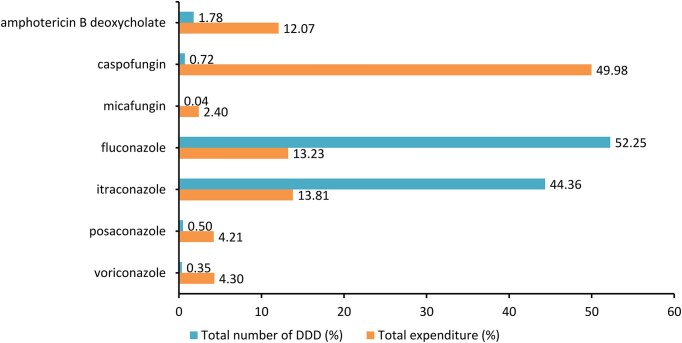
Proportion of the total defined daily doses (DDD) and expenditure of each antifungal agent in 5 years (from 2018 to 2022).

The most consumed antifungals in Vietnam were fluconazole (52.25%) and itraconazole (44.36%), followed by amphotericin B deoxycholate (1.78%), caspofungin (0.72%), posaconazole (0.50%), voriconazole (0.35%), and micafungin (0.04%) (Fig. 2). Caspofungin was the most expensive antifungal agent. Despite accounting for less than 1% of DDD, caspofungin contributed to 50% of the total antifungal expenditure, which was 28.8 million USD in 5 years. In contrast, the most used antifungal agents, fluconazole and itraconazole, only accounted for 13.2% and 13.8% of the total expenditure, respectively. Amphotericin B deoxycholate contributed a similar proportion of the total expenditure (12.1%) despite its low number of DDD of less than 2% in comparison to the azoles.

### Sources of antifungals in Vietnam

A large proportion of antifungals consumed in Vietnam (68.2%) were manufactured domestically but represented only 8% of the total antifungal expenditure in 5 years. Besides, this portion of antifungals only consisted of azole antifungals. Vietnam relied on other countries for the supply of amphotericin B deoxycholate and echinocandins.

India was the largest provider of antifungals to Vietnam, supplying 100% of amphotericin B deoxycholate, 31.1% of echinocandins, and 7.7% of azole antifungals. Echinocandins were imported from three countries, with France being the largest provider (63.2% of DDD), followed by India (31.1% of DDD) and Japan (5.7% of DDD). Vietnam manufactured most of the azole antifungals domestically (70%). The other 30% of azole antifungals were imported internationally, consisting of 8.9% from Romania (a high-income country), 7.7% from India (a lower-middle-income country), 2.8% from Thailand (an upper-middle-income country), and the remaining 10.6% from 17 other countries (including 12 high-income countries, three upper-middle-income countries, and two lower-middle-income countries) (Supplementary Table 5). Each of these 17 countries supplied less than 2% of the azole antifungals in Vietnam.

Notably, we noticed disproportionately high costs of azole antifungals from three high-income countries (Italy, Canada, and France) and one lower-middle-income country (India) relative to their numbers of DDD imported. Italy, Canada, and France supplied 0.9%, 0.5%, and 0.4% of the azole antifungals each, which made up 12%, 11.9%, and 5.6% of the expenditure in this antifungal class, respectively. In contrast, the 70% of locally manufactured azole antifungals only contributed to 22.4% of the cost spent on this class of antifungals. The 14.3% of the expenditure spent on importing 7.7% of the azole antifungals from India, a lower-middle-income country, may seem reasonable at first in comparison; however, it is in fact more expensive than that of Romania, a high-income country, which supplied 8.9% of the azole derivatives with just 10.1% of the expenditure in this class of antifungal.

### Cost per DDD of antifungals in Vietnam

We summarised the average cost per DDD of each antifungal agent in Table 2. Echinocandins were the most expensive class of antifungals in Vietnam. A DDD of caspofungin and micafungin costs 257 USD and 209 USD, respectively. Fluconazole and itraconazole were the two cheapest antifungals, costing 4 USD and 7 USD per DDD each. On the other hand, the other two azoles, posaconazole and voriconazole, were rather expensive, costing 31 USD and 55 USD per DDD, respectively.

**Table 2.** Average cost (in United States dollar, USD) per defined daily dose (DDD) of antifungal agents purchased by Vietnam from different manufacturing countries.*

	Manufacturing country				
Antifungal agent	High-income	Upper-middle income	Lower-middle income	Vietnam	Average cost per DDD for each antifungal
**Polyene**					
Amphotericin B deoxycholate	–	–	$23.64	–	$23.64
**Echinocandins**					
Caspofungin	$285.20	–	$189.15	–	$257.08
Micafungin	$208.55	–	–	–	$208.55
**Azole derivatives**					
Fluconazole	$9.41	$2.11	$5.14	$0.41	$4.09
Itraconazole	$24.70	$1.88	$0.68	$0.56	$7.43
Posaconazole	$31.00		–	–	$31.00
Voriconazole	–	–	$60.44	$33.16	$54.98
**Average cost per DDD for each group of countries**	$96.76	$1.96	$50.96	$0.89	$46.49

$, United States dollar (USD); DDD, defined daily dose

* The prices in the table are not rounded whereas the prices listed in the text have been rounded to the nearest USD.

In general, the cost per DDD of an antifungal was the cheapest when locally manufactured in comparison to that of imported antifungals (Table 2). The cost was also cheaper when purchased from a lower-middle-income country instead of a high-income country. For instance, the cost per DDD of imported voriconazole (60 USD) was nearly double the cost of locally manufactured voriconazole (33 USD). The cost per DDD of itraconazole imported from high-income countries (25 USD) was 36 times higher than that imported from lower middle-income countries (0.70 USD) and 44 times higher than that produced in Vietnam (0.60 USD).

### Cost of antifungal treatment for IFIs in Vietnam

Table 3 summarises the costs of antifungal treatments for invasive candidiasis and candidaemia, invasive pulmonary aspergillosis, cryptococcal meningitis, and talaromycosis. As we were unable to estimate the actual number of cases that were treated by first versus alternative lines of treatment, the costs of each line of treatment displayed in Table 3 were the costs that would have been incurred if all cases of an IFI in 2020 had been diagnosed and solely treated using that line of regimen.

**Table 3.** Cost (in United States dollar, USD) of antifungal treatment in Vietnam.,^**,†^

Fungal species (main IFI/s)	Regimen	Daily dosage	Duration	Cost per course (USD)	Total cost based on estimated disease burden in 2020 (USD)^7^
*Candida* (invasive candidiasis/candidaemia)	**First line**				
	Caspofungin	Loading: 70 mg Maintenance: 50 mg	2 weeks^‡^	$3701.95	$41.8 million
	Micafungin	100 mg	2 weeks^‡^	$2919.70	$33 million
	Fluconazole (non-critically ill)	Loading: 800 mg Maintenance: 400 mg	2 weeks^‡^	$122.70	$1.4 million
	**Second line (intolerance/resistance to other antifungals)**				
	Amphotericin B deoxycholate^¶^	49–70 mg	2 weeks^‡^	$463.34–$661.92	$5.2 million–$7.5 million
*Aspergillus* (invasive pulmonary aspergillosis)	**First line**				
	Voriconazole (preferred)	Loading: IV 840 mg or PO 800 mg on day 1 Maintenance: IV 560 mg or PO 400–600 mg	6–12 weeks	IV: $3271.31–$6504.13 PO: $2364.14–$6954.97	IV: $76.8 million–$152.7 million PO: $55.5 million–$163.2 million
	Amphotericin B deoxycholate^¶^ (alternative)	49–70 mg	6–12 weeks	$1390.20–$3971.52	$32.6 million–$93.2 million
	**Second line/other options**				
	Caspofungin	Loading: 70 mg Maintenance: 50 mg	6–12 weeks	$10 900.19–$21 697.55	$255.8–509.2 million
	Micafungin	100–150 mg	6–12 weeks	$8759.10–$26 277.30	$205.6–616.7 million
	Posaconazole	600 mg	6–12 weeks	$2604–$5208	$61.1–122.2 million
	Itraconazole	400 mg	6–12 weeks	$624.12–$1248.24	$14.6–29.3 million
*Cryptococcus* (Cryptococcal meningitis)^#^	**Induction**				
	Amphotericin B deoxycholate + fluconazole	49–70 mg + 800 mg	2 weeks	$692.44–$890.96	$0.3–0.4 million
	Amphotericin B deoxycholate	49–70 mg	2 weeks	$463.40–$661.92	$0.2–0.3 million
	Fluconazole	1200 mg	2 weeks	$343.56	$0.2 million
	**Consolidation**				
	Fluconazole	400 mg	8 weeks	$458.08	$0.2 million
	Itraconazole	400 mg	8 weeks	$832.16	$0.4 million
	**Maintenance**				
	Fluconazole	200 mg	1 year	$1492.85	$0.7 million
*Talaromyces marneffei* (talaromycosis)^§^	**First line**				
	Amphotericin B deoxycholate^¶^ (induction)	49–70 mg	2 weeks	$463.40–$661.92	$0.7 million–$1.1 million
	Itraconazole (maintenance)	400 mg	10 weeks	$1040.20	$1.7 million
	**Alternative**				
	Voriconazole (induction)	Loading: IV 840 mg on day 1 Maintenance: IV 560 mg	4 days	$346.38	$0.6 million
	Voriconazole (maintenance)	PO 400 mg	12 weeks	$4618.32	7.4 million

IFI, invasive fungal infection; USD/$, United States dollar; IV, intravenous; PO, per oral.

* As per WHO guideline, the DDD is defined as 35 mg for amphotericin B deoxycholate, 50 mg for caspofungin, 200 mg for fluconazole, 200 mg for itraconazole, 100 mg for micafungin, 300 mg for posaconazole and 400 mg for voriconazole.

** Weight used to calculate dose: 70 kg.

† The costs listed in the table are not rounded whereas the costs in the text have been rounded to the nearest USD.

‡ Assumed a 14-day course.

¶ In the absence of an antifungal agent recommended by the Guidelines for the Diagnosis and Treatment of Invasive Fungal Infections 2021, prescribers may choose alternatives that are available in Vietnam.^9^ This calculation assumes prescribers substitute liposomal amphotericin B, which is not available in Vietnam, with amphotericin B deoxycholate (the only formulation of amphotericin B that is available in Vietnam) when appropriate.

# In patients who are HIV positive or persistently immunocompromised.

§ Moderate to severe infection: damage to multiple organs without (moderate)/with (severe) respiratory or circulatory failure.

A first-line treatment for invasive candidiasis and candidaemia using caspofungin would incur 3702 USD in a 2-week course of treatment. The actual duration of treatment in practice may be longer, as the recommended duration is until 2 weeks after blood cultures have shown negative results and clinical improvement in symptoms are observed.^9^ However, echinocandin and amphotericin B deoxycholate may be transitioned to high-dose fluconazole (800 mg daily) if the *Candida* species is susceptible to fluconazole, the subsequent blood culture returns negative, and the patient is clinically stable.^9^ In non-critically ill patients, physicians may opt to prescribe fluconazole as a relatively cheaper first-line treatment if the infection is fluconazole-susceptible, reducing the cost per course to 123 USD.

For invasive aspergillosis, the preferred first-line treatment using voriconazole would cost up to 6955 USD for a 12-week course.

The treatment of cryptococcal meningitis involves induction, consolidation, and maintenance therapy. A combined course of amphotericin B deoxycholate and fluconazole for induction, fluconazole for consolidation and maintenance, takes 62 weeks and costs up to 2842 USD.

Treatment of moderate to severe *Talaromyces marneffei* infection using amphotericin B deoxycholate for induction and itraconazole for maintenance would cost up to 1702 USD in a 12-week course, whereas treatment with voriconazole for both induction and maintenance would cost 4965 USD per 12-week-and-4-day course.

Vietnam was estimated to have 11 291 cases of candidaemia, 23 470 cases of invasive aspergillosis, 451 cases of cryptococcal meningitis, and 1612 cases of talaromycosis in 2020.^7^ Assuming that all cases were identified, and received a full course of first-line therapy (i.e., there were no deaths or loss to follow-up), Vietnam would have spent up to 41.8 million USD on the treatment of candidaemia, up to 163.2 million USD on treating invasive aspergillosis, 1.3 million USD on treating cryptococcal meningitis, and up to 2.8 million USD on treating talaromycosis. The estimated financial burden of antifungal treatment in Vietnam on four IFIs alone would have been a total of 209.1 million USD in pharmacological treatment alone in 2020.

## Discussion

### Key findings

This study revealed that the following antifungal agents are available in Vietnam for the therapy of IFIs as of 2022: amphotericin B deoxycholate, fluconazole, itraconazole, voriconazole, caspofungin, and micafungin (listed by the WHO on the EML), as well as posaconazole. The most consumed antifungal agents are fluconazole and itraconazole, whereas the most expensive antifungal agent is caspofungin. While domestically manufactured antifungal agents are cheaper in cost in comparison to agents manufactured in foreign countries, Vietnam only manufactured the majority of the azole antifungals and relied on imports for the supply of amphotericin B deoxycholate and echinocandins. We also identified invasive aspergillosis as the biggest financial burden to the country in terms of IFI pharmacotherapy.

### Availability of WHO EML antifungal agents

Vietnam has access to all antifungal agents on the WHO EML for systemic infections except flucytosine. In a 2013 trial in Vietnam and a 2018 trial in Africa, flucytosine was shown to be superior to fluconazole in combination induction therapy with amphotericin B in reducing the mortality rate of cryptococcal meningitis among patients with HIV.^15,16^ The study in Vietnam led to the reinstatement of flucytosine on the EML by the WHO in 2013.^17^ However, our study showed that flucytosine remained unavailable in Vietnam, consistent with the findings of previous studies that showed similar situations in many South-East Asian countries, and the high cost was likely a contributory factor.^4,18^

### Most and least consumed antifungal agents in Vietnam versus other countries

A 2022 study on the global use of antifungal medications covering 27 middle-income and 38 high-income nations between 2008 and 2018 has identified azole antifungals such as itraconazole and fluconazole to be among the top three most consumed antifungal agents in middle- and high-income countries in 2018.^5^ On the other hand, polyenes and echinocandins were the least used antifungal agents.^5^ These findings were consistent with our study. Similar to Lebanon, also a middle-income country like Vietnam, our study identified fluconazole as the most used antifungal, while micafungin was the least consumed antifungal agent.^19^

### Cost—domestic versus foreign manufacturers

The 2016 study by Kneale et al. reported the price of amphotericin B in Vietnam to be 13.76 USD (50 mg/dose), fluconazole to be less than 1 USD (750–800 mg/dose), and itraconazole to be less than 1 USD (400 mg/dose).^4^ Our study found the price of amphotericin B (in the form of deoxycholate) to be more expensive (23.64 USD/35 mg dose), whereas the price of less than 1 USD for fluconazole (200 mg/dose) and itraconazole (200 mg/dose) was only possible if purchased locally (fluconazole, itraconazole) or from lower-middle-income countries (itraconazole).

Domestically manufactured antifungals were found to be cheaper than imported antifungals. However, the local sector remained under-explored, with azole antifungals being the only antifungal class manufactured domestically and only contributing to 70% of the azole antifungal consumption in the country. Vietnam relied heavily on imported antifungal medications, including purchasing from countries with higher prices. For example, caspofungin from high-income countries was more expensive than those from lower-middle-income countries. However, the purchase of caspofungin from France, a high-income country, was over double that from India, a lower-middle-income country (63.2% versus 31.1% of the total purchase).

The fluctuation in the number of DDD and expenditure of a drug seen in our study correlates with the fluctuation in the number of procurement units which submitted their data over the years, as well as the proportion of data from centralised bidding or hospitals of higher tiers versus lower tiers each year. We observed that centralised bidding and hospitals of higher tiers tended to order larger quantities of DDD compared to hospitals of lower tiers. The fluctuation over the years is also influenced by the proportion of purchases from high-income countries versus upper-middle- or lower-middle-income countries each year. Our study showed that any shift in sources of purchase could impact the price and DDD in a year, highlighting the importance of cost-effective procurement.

### Pharmacotherapy cost of IFIs in Vietnam

IFIs caused a huge economic burden to Vietnam. The potential maximum financial burden of 209.1 million USD estimated for the pharmacological treatment of four IFIs alone in 2020 would be incurred if all cases were identified and treated with first line of treatment, and no patients died or were lost to follow up.^7^ It is therefore an overestimate, but is nonetheless already very significantly higher than the available data on the antifungal expenditure in 2020 (at least 7.4 million USD), and indicates that costs could rise rapidly with increased diagnoses, improved reporting of procurement data and the inclusion of other IFI diagnoses in the calculation. The full economic burden would have been greater than 7.4 million USD, as treatment involves non-pharmaceutical costs such as specialised staffing, facility and equipment, hospitalisation fees, especially intensive care unit stays, etc. A study by Benedict et al. has estimated the direct medical cost of fungal diseases to be 7.5 billion USD in the United States of America in 2019.^20^*Candida* infections (invasive and non-invasive) and *Aspergillus* infections were found to be the greatest contributors, accounting for 3.4 billion USD and 1.3 billion USD, respectively.^20^ Our study found that invasive aspergillosis would have accounted for the highest pharmaceutical costs (163.2 million USD) in Vietnam due to its high disease burden. While first-line medications may not always be used in resource-limited countries when the agents are expensive, switching first-line antifungals to second-line agents may be associated with prolonged hospital and intensive care unit length of stay, which subsequently contributes to additional costs that undermine cost savings from cheaper antifungal agents.^21^

### Availability of procurement data

In Vietnam, hospitals are mandated to publish their procurement data annually but are given the liberty to publish on any platform of their choice, including internal websites that are not accessible to the public and any mass media sites. This inconsistency in the publication site not only creates intransparency due to difficulty in searching up the purchasing figures for public scrutiny but also causes enormous hurdles for the annual consumption and expenditure of the country to be analysed accurately. Additionally, the format of the submitted data from each procurement unit lacks consistency, with some data published in Excel spreadsheets and different full or abbreviated names used by each site. We propose introducing a standardised publication format of the annual procurement amount and expenditure in a single and official source, i.e., the Drug Administration of Vietnam website, for ease of public view and comparison.

### Limitations

There are some limitations in this study. First, as procurement data were used, the exact consumption might be different. Second, the actual expenditure and consumption of the country would have been underestimated, as only publicly published data on the Drug Administration of Vietnam website was collected, and hospitalised patients could directly purchase the medications if the medications were unavailable in hospital pharmacies.^11^ Data from internal institutional websites, mass media, private hospitals, and private out-of-pocket purchases by patients would have been omitted. Third, the trend of expenditure and consumption over the years could not be analysed, as the total expenditure and consumption for a given year is largely driven by the number and tiers of procurement units that reported their data to the Drug Administration of Vietnam in that year (Supplementary Table 4). Lastly, the actual financial burden might be different from estimation, as it was difficult to estimate the proportion of cases treated by the first versus alternative lines of treatment.

### Future perspectives

Our study shows that changes in clinical or procurement behaviours could have profound impacts on costs. Antifungal stewardship, intravenous to oral switch when appropriate, public health policies targeting the prevention of diseases such as HIV/AIDS and tuberculosis that put patients at high risk of IFIs, and sourcing from cheaper manufacturers may help reduce costs and subsequently ensure sustainable access across the country. The local antifungal pharmaceutical industry should be strengthened in line with the Vietnam National Strategy for Protection, Care and Improvement of the People’s Health by 2030 with a Vision towards 2045, which aims to prioritise the development of local pharmaceuticals.^22^ There may also be a need to reassess the sustainability of the current method of purchasing and prescribing practice, including ensuring fair and transparent bidding processes, accrediting and reinforcing good manufacturing practice in local manufacturers to gain public trust, and correcting misconceptions that the lower costs of generic medications imply they are of lower quality than brand originators.^23–25^

Future studies should investigate the guideline compliance and appropriateness of antifungal prescribing practice in both treatment and prophylaxis and identify the potential cost saving or discrepancy if antifungals are prescribed appropriately in Vietnam. Further cost-effectiveness studies are also required in lower middle-income countries like Vietnam to understand the value of investing in expensive first-line medications such as flucytosine and liposomal amphotericin B.^26,27^

## Conclusion

Vietnam has access to all the antifungal agents listed on the WHO EML for systemic fungal infections except flucytosine. The most consumed antifungal agents are fluconazole and itraconazole. Antifungal agents for IFIs are a major burden on the healthcare system of Vietnam. The financial burden was mainly contributed to by the heavy disease burden caused by aspergillosis, expensive agents such as echinocandins and dependence on expensive imported antifungal agents.

## Supplementary Material

myaf028_Supplemental_File

## Data Availability

The data that support the findings of this study are available from the corresponding author upon reasonable request.
