# Supplementary material for: Availability and cost of antifungal therapy in Vietnam: A 5-year retrospective study
Source: Med Mycol. 2025 Apr 1;63(4):myaf028. doi: 10.1093/mmy/myaf028 (PMC12001882; doi:10.1093/mmy/myaf028)
Supplement: myaf028_Supplemental_File [file myaf028_supplemental_file.docx]

**Supplemental Content**

**Table S1.** The number of procurement units with at least three years of data reported for each antifungal agent out of 261 procurement units investigated.

| **Antifungal agents** | **Number of procurement units with ≥3 years of data** | **Number of procurement units with ≥4 years of data** | **Number of procurement units with 5 years of data** |
| --- | --- | --- | --- |
| Amphotericin B deoxycholate | 23 | 8 | 1 |
| Caspofungin | 25 | 10 | 2 |
| Fluconazole | 84 | 33 | 4 |
| Itraconazole | 78 | 27 | 5 |
| Micafungin | 0 | 0 | 0 |
| Posaconazole | 2 | 3 | 0 |
| Voriconazole | 5 | 0 | 0 |

**Table S2.** Expenditure (in United States dollar, USD) and number of defined daily dose (DDD) of antifungal agents procured at different tiers of hospitals in Vietnam from 2018 to 2022.

|  | | | 2018 | 2019 | 2020 | 2021 | 2022 |
| --- | --- | --- | --- | --- | --- | --- | --- |
| Amphotericin B deoxycholate | Centralised bidding | Expenditure | 37,697.83 | 76,847.76 | 27,577.00 | 70,411.13 | 69,282.46 |
|  |  | Number of DDD | 6,095.71 | 4,594.29 | 5,300.00 | 5,385.71 | 11,255.71 |
|  | National hospitals | Expenditure | 1,619,915.87 | 1,096,760.70 | 683,612.32 | 691,594.24 | 773,723.20 |
|  |  | Number of DDD | 50,731.43 | 26,714.29 | 22,235.71 | 18,748.57 | 21,300.00 |
|  | Provincial hospitals | Expenditure | 393,290.24 | 245,304.70 | 123,255.36 | 207,344.62 | 788,745.62 |
|  |  | Number of DDD | 29,114.29 | 10,664.29 | 9,314.29 | 15,510.00 | 37,282.86 |
|  | District hospitals | Expenditure | 4,463.75 | 20,333.57 | 2,459.37 | 20,545.07 | N/A |
|  |  | Number of DDD | 71.43 | 600.00 | 42.86 | 622.86 | N/A |
| Caspofungin | Centralised bidding | Expenditure | N/A | 325,097.84 | 105,043.66 | 346,156.97 | 269,319.52 |
|  |  | Number of DDD | N/A | 1,063.20 | 357.60 | 1,287.00 | 1,172.00 |
|  | National hospitals | Expenditure | 3,383,659.74 | 2,402,950.38 | 2,879,352.96 | 5,120,029.07 | 7,374,566.32 |
|  |  | Number of DDD | 10,675.00 | 8,764.00 | 10,983.60 | 21,041.60 | 30,526.80 |
|  | Provincial hospitals | Expenditure | 1,307,826.31 | 728240.34 | 660,328.22 | 1,509,417.51 | 2,215,270.35 |
|  |  | Number of DDD | 4,138.40 | 2,418.00 | 2,360.00 | 5,913.60 | 9,510.00 |
|  | District hospitals | Expenditure | N/A | 88,217.86 | 27,570.78 | 49,478.76 | N/A |
|  |  | Number of DDD | N/A | 288.00 | 94.00 | 202.00 | N/A |
| Fluconazole | Centralised bidding | Expenditure | 1,199,279.60 | 587,693.75 | 590,895.16 | 619,956.33 | 476,630.55 |
|  |  | Number of DDD | 2,205,189.00 | 948,245.00 | 681,373.00 | 1,093,788.00 | 735,346.50 |
|  | National hospitals | Expenditure | 901,651.78 | 497,435.23 | 725,321.52 | 422,064.35 | 173,072.84 |
|  |  | Number of DDD | 263,029.00 | 128,440.00 | 233,970.00 | 133,267.50 | 57,090.00 |
|  | Provincial hospitals | Expenditure | 511,866.35 | 218,296.08 | 100,503.42 | 264,188.84 | 132,600.26 |
|  |  | Number of DDD | 554,200.50 | 167,018.75 | 85,000.50 | 224,249.50 | 263,817.75 |
|  | District hospitals | Expenditure | 13,972.35 | 113,090.46 | 44,443.14 | 18,349.37 | 11,277.83 |
|  |  | Number of DDD | 23,165.00 | 111,900.00 | 96,385.00 | 48,772.00 | 28,454.00 |
| Itraconazole | Centralised bidding | Expenditure | 1,924,204.94 | 741,887.16 | 343,042.04 | 559,495.99 | 229,221.81 |
|  |  | Number of DDD | 1,888,192.50 | 907,300.50 | 566,127.00 | 954,612.50 | 329,046.00 |
|  | National hospitals | Expenditure | 1,392,447.27 | 262,932.05 | 348,186.52 | 87,910.83 | 131,179.16 |
|  |  | Number of DDD | 334,877.50 | 88,187.50 | 134,702.50 | 80,000.00 | 132,470.00 |
|  | Provincial hospitals | Expenditure | 1,044,673.75 | 270,989.75 | 153,050.47 | 106,457.09 | 124,912.11 |
|  |  | Number of DDD | 512,775.75 | 160,425.00 | 129,475.50 | 196,865.00 | 198,308.00 |
|  | District hospitals | Expenditure | 53,700.70 | 53,591.30 | 47,005.19 | 64,500.03 | 17,262.75 |
|  |  | Number of DDD | 37,800.00 | 42,150.00 | 61,000.00 | 68,800.00 | 39,400.00 |
| Micafungin | Centralised bidding | Expenditure | N/A | N/A | N/A | 28,906.62 | 245,329.42 |
|  |  | Number of DDD | N/A | N/A | N/A | 135.00 | 1,195.00 |
|  | National hospitals | Expenditure | N/A | N/A | N/A | 128,366.83 | 548,655.13 |
|  |  | Number of DDD | N/A | N/A | N/A | 599.50 | 2,672.50 |
|  | Provincial hospitals | Expenditure | N/A | N/A | N/A | 89,824.66 | 343,871.78 |
|  |  | Number of DDD | N/A | N/A | N/A | 419.50 | 1,675.00 |
|  | District hospitals | Expenditure | N/A | N/A | N/A | N/A | N/A |
|  |  | Number of DDD | N/A | N/A | N/A | N/A | N/A |
| Posaconazole | Centralised bidding | Expenditure | N/A | N/A | N/A | N/A | N/A |
|  |  | Number of DDD | N/A | N/A | N/A | N/A | N/A |
|  | National hospitals | Expenditure | 565,408.89 | 647,493.48 | 432,667.18 | 302,304.69 | 260,042.34 |
|  |  | Number of DDD | 16,800.00 | 20,160.00 | 14,000 | 9,940.00 | 8,918.00 |
|  | Provincial hospitals | Expenditure | 94,234.81 | N/A | 51,920.06 | N/A | 74,297.81 |
|  |  | Number of DDD | 2,800.00 | N/A | 1,680.00 | N/A | 2,548.00 |
|  | District hospitals | Expenditure | N/A | N/A | N/A | N/A | N/A |
|  |  | Number of DDD | N/A | N/A | N/A | N/A | N/A |
| Voriconazole | Centralised bidding | Expenditure | N/A | N/A | 18,080.93 | 57,973.48 | N/A |
|  |  | Number of DDD | N/A | N/A | 500.00 | 1,150.00 | N/A |
|  | National hospitals | Expenditure | 196,901.17 | 640,867.08 | 68,580.03 | 290,021.57 | 765,394.24 |
|  |  | Number of DDD | 5,000.00 | 17,550.00 | 1,200.00 | 5,460.00 | 14,960.00 |
|  | Provincial hospitals | Expenditure | 32,957.39 | 31,451.71 | 12,911.70 | 86,478.41 | 273,272.00 |
|  |  | Number of DDD | 500.00 | 500.00 | 150.00 | 1,750.00 | 5,260.00 |
|  | District hospitals | Expenditure | N/A | N/A | N/A | N/A | N/A |
|  |  | Number of DDD | N/A | N/A | N/A | N/A | N/A |
| Total of all antifungals | | **Expenditure (%)** | **14,678,152.75 (100.00%)** | **9,049,481.20**  **(100.00%)** | **7,445,807.03**  **(100.00%)** | **11,141,776.46**  **(100.00%)** | **15,297,927.50**  **(100.00%)** |
|  |  | **Number of DDD (%)** | **5,945,155.51 (100.00%)** | **2,646,982.81 (100.00%)** | **2,056,251.56 (100.00%)** | **2,888,519.84 (100.00%)** | **1,932,208.12 (100.00%)** |

DDD: defined daily dose; N/A: data was not available.

**Table S3.** The number of procurement unit with at least three consecutive years of data out of the 261 procurement units investigated.

| **Antifungal agents** | **Number of procurement units** |
| --- | --- |
| Amphotericin B | 6 |
| Caspofungin | 7 |
| Fluconazole | 29 |
| Itraconazole | 32 |
| Micafungin | 0 |
| Posaconazole | 1 |
| Voriconazole | 0 |

**Table S4.** The number of procurement units which submitted procurement data of antifungal agents to the Drug Administration of Vietnam website from 2018 – 2022.

| **Antifungal agents** | **Tier of hospitals** | **2018** | **2019** | **2020** | **2021** | **2022** |
| --- | --- | --- | --- | --- | --- | --- |
| Amphotericin | Centralised bidding | 11 | 7 | 5 | 6 | 15 |
|  | National hospitals | 14 | 9 | 11 | 8 | 7 |
|  | Provincial hospitals | 18 | 13 | 8 | 15 | 15 |
|  | District hospitals | 1 | 1 | 1 | 2 | 0 |
| Caspofungin | Centralised bidding | 0 | 1 | 2 | 4 | 7 |
|  | National hospitals | 8 | 7 | 7 | 12 | 15 |
|  | Provincial hospitals | 13 | 11 | 3 | 26 | 21 |
|  | District hospitals | 0 | 1 | 2 | 3 | 0 |
| Fluconazole | Centralised bidding | 45 | 22 | 21 | 28 | 30 |
|  | National hospitals | 22 | 14 | 17 | 15 | 18 |
|  | Provincial hospitals | 51 | 26 | 21 | 47 | 24 |
|  | District hospitals | 9 | 10 | 25 | 19 | 9 |
| Itraconazole | Centralised bidding | 47 | 21 | 20 | 24 | 23 |
|  | National hospitals | 25 | 13 | 12 | 9 | 15 |
|  | Provincial hospitals | 37 | 23 | 25 | 32 | 41 |
|  | District hospitals | 5 | 10 | 14 | 18 | 20 |
| Micafungin | Centralised bidding | 0 | 0 | 0 | 1 | 6 |
|  | National hospitals | 0 | 0 | 0 | 7 | 8 |
|  | Provincial hospitals | 0 | 0 | 0 | 4 | 9 |
|  | District hospitals | 0 | 0 | 0 | 0 | 0 |
| Posaconazole | Centralised bidding | 0 | 0 | 0 | 0 | 0 |
|  | National hospitals | 3 | 2 | 1 | 3 | 5 |
|  | Provincial hospitals | 1 | 0 | 1 | 0 | 3 |
|  | District hospitals | 0 | 0 | 0 | 0 | 0 |
| Voriconazole | Centralised bidding | 0 | 0 | 1 | 3 | 0 |
|  | National hospitals | 3 | 2 | 1 | 4 | 7 |
|  | Provincial hospitals | 1 | 1 | 1 | 2 | 7 |
|  | District hospitals | 0 | 0 | 0 | 0 | 0 |
| Total data submission | | 314 | 194 | 199 | 292 | 305 |

**Table S5.** Sources of antifungal agents in Vietnam.^a^

| Manufacturing country | Amphotericin B deoxycholate | | Echinocandins | | Azole antifungals | | All antifungals | |
| --- | --- | --- | --- | --- | --- | --- | --- | --- |
|  | Number of DDD (%) | Expenditure (%) | Number of DDD (%) | Expenditure (%) | Number of DDD (%) | Expenditure (%) | Total Number of DDD (%) | Total Expenditure (%) |
| High-income countries | | | | | | | | |
| Canada | 0.00 | 0.00 | 0.00 | 0.00 | 0.51 | 11.86 | 0.50 | 4.21 |
| Cyprus | 0.00 | 0.00 | 0.00 | 0.00 | 1.59 | 2.01 | 1.55 | 0.72 |
| France | 0.00 | 0.00 | 63.22 | 71.71 | 0.43 | 5.57 | 0.90 | 39.54 |
| Germany | 0.00 | 0.00 | 0.00 | 0.00 | 0.44 | 3.51 | 0.43 | 1.25 |
| Greece | 0.00 | 0.00 | 0.00 | 0.00 | 0.54 | 3.65 | 0.53 | 1.30 |
| Hungary | 0.00 | 0.00 | 0.00 | 0.00 | 0.32 | 2.79 | 0.31 | 0.99 |
| Italy | 0.00 | 0.00 | 0.00 | 0.00 | 0.87 | 11.99 | 0.85 | 4.26 |
| Japan | 0.00 | 0.00 | 5.70 | 4.59 | 0.00 | 0.00 | 0.04 | 2.40 |
| Poland | 0.00 | 0.00 | 0.00 | 0.00 | 0.36 | 0.31 | 0.35 | 0.11 |
| Romania | 0.00 | 0.00 | 0.00 | 0.00 | 8.87 | 10.05 | 8.65 | 3.57 |
| Slovenia | 0.00 | 0.00 | 0.00 | 0.00 | 1.16 | 1.38 | 1.13 | 0.49 |
| South Korea | 0.00 | 0.00 | 0.00 | 0.00 | 1.52 | 1.08 | 1.49 | 0.38 |
| Spain | 0.00 | 0.00 | 0.00 | 0.00 | 0.19 | 0.19 | 0.19 | 0.07 |
| Taiwan | 0.00 | 0.00 | 0.00 | 0.00 | 0.05 | 0.22 | 0.05 | 0.08 |
| Upper middle-income countries | | | | | | | | |
| Belarus | 0.00 | 0.00 | 0.00 | 0.00 | 0.03 | 0.19 | 0.03 | 0.07 |
| Bulgaria | 0.00 | 0.00 | 0.00 | 0.00 | 1.04 | 1.23 | 1.01 | 0.44 |
| China | 0.00 | 0.00 | 0.00 | 0.00 | 0.04 | 0.11 | 0.03 | 0.04 |
| Thailand | 0.00 | 0.00 | 0.00 | 0.00 | 2.79 | 3.86 | 2.72 | 1.37 |
| Lower middle-income countries | | | | | | | | |
| India | 100.00 | 100.00 | 31.08 | 23.70 | 7.72 | 14.31 | 9.54 | 29.57 |
| Indonesia | 0.00 | 0.00 | 0.00 | 0.00 | 1.03 | 0.86 | 1.00 | 0.31 |
| Ukraine | 0.00 | 0.00 | 0.00 | 0.00 | 0.48 | 2.46 | 0.47 | 0.87 |
| Vietnam | 0.00 | 0.00 | 0.00 | 0.00 | 70.02 | 22.37 | 68.24 | 7.95 |
| Grand total | **100.00** | **100.00** | **100.00** | **100.00** | **100.00** | **100.00** | **100.00** | **100.00** |

DDD: defined daily dose

^a^ The high-income, upper middle-income and lower middle-income countries were classified in accordance with the World Bank classification system 2022.
